# Multiscale analysis of the hydrate based carbon capture from gas mixtures containing carbon dioxide

**DOI:** 10.1038/s41598-021-88531-x

**Published:** 2021-04-28

**Authors:** Xuebing Zhou, Xiaoya Zang, Zhen Long, Deqing Liang

**Affiliations:** 1 Guangzhou Institute of Energy Conversion, Chinese Academy of Sciences, Guangzhou, 510640 China; 2 CAS Key Laboratory of Gas Hydrate, Guangzhou, 510640 China; 3 Guangdong Provincial Key Laboratory of New and Renewable Energy Research and Development, Guangzhou, 510640 China; 4 State Key Laboratory of Natural Gas Hydrate, Beijing, 100028 China

**Keywords:** Chemical physics, Engineering, Chemical engineering, Energy science and technology, Carbon capture and storage, Energy storage

## Abstract

To reveal the kinetic performance of gas molecules in hydrate growth, hydrate formation from pure CO_2_, flue gas, and biogas was measured using in-situ Raman and macroscopic methods at 271.6 K. In the in-situ Raman measurements, Raman peaks of gases in the hydrate phase were characterised and normalised by taking the water bands from 2800 to 3800 cm^−1^ as a reference, whose line shapes were not found to have a noticeable change in the conversion from Ih ice to sI hydrate. The hydrate growth was suggested to start with the formation of unsaturated hydrate nuclei followed by gas adsorption. In hydrate formed from all tested gases, CO_2_ concentrations in hydrate nuclei were found to be 23–33% of the saturation state. In the flue gas system, the N_2_ concentration reached a saturation state once hydrate nuclei formed. In the biogas system, competitive adsorption of CH_4_ and CO_2_ molecules was observed, while N_2_ molecules hardly evolved in hydrate formation. Combined with micro- and macroscopic analysis, small molecules such as N_2_ and CO_2_ were suggested to be more active in the formation of hydrate nuclei, and the preferential adsorption of CO_2_ molecules took place in the subsequent gas adsorption process.

## Introduction

Gas hydrates are ice-like crystalline minerals where gas molecules are trapped in the hydrogen-bonded network with distinct polyhedral water cages, and they typically form at ambient temperature and moderate pressure^1^. Gas hydrates have gained global attention as a potential energy source owing to their wide distribution in the permafrost and ocean floor^2^. They have also been considered as eco-friendly and energy-saving materials for carbon capture and sequestration (CCS)^3^. In this case, understanding the kinetic properties of gas hydrates is essential to the successful applications of gas production from hydrate-bearing deposits and CCS technologies.

The preferential incorporation of CO_2_ in hydrate formation from gas mixtures is key to hydrate-based CCS technologies. Usually, the gas mixtures containing CO_2_ form typical sI hydrates, which include six tetrakaidecahedron (5^12^6^2^) cages and two pentagonal dodecahedron (5^12^) cages per unit cell. Structural analysis has revealed that high gas occupancies in the large (5^12^6^2^) cages are important for stabilising sI hydrate^4,5^. CO_2_ molecules have a size capable of supporting the 5^12^6^2^ cage structure, while N_2_, CH_4_, and H_2_ molecules are all slightly too small^6–8^. This allows CO_2_ molecules to be enriched in the hydrate phase. Gas hydrates are thus proposed to adsorb CO_2_ from flue gas (N_2_/CO_2_), precombustion gas (H_2_/CO_2_) from integrated gasification combined cycles, or contaminated natural gas (CH_4_/CO_2_) in power plants^9–11^.

Based on thermodynamic analysis, investigations of hydrate-based carbon capture have been divided into three methodological categories. The first is the enhancement of the separation efficiency by lowering the stability of formed hydrates. In harsh environments, more CO_2_ molecules are adsorbed in the hydrate phase to increase hydrate stability. Therefore, increasing the temperature or adding solutes such as inorganic salts are common methods of enhancing the selectivity of formed hydrates^12,13^. The second method is to allow hydrate to form at milder running conditions during crystallisation. Hydrate promoters such as tetrahydrofuran and ionic salts allow hydrates to form at about 275 K and atmospheric pressure^14–16^. The last method is to strengthen the gas adsorption rate of the hydrates. Hydrate formation on porous materials or in stirring reactors is proposed to enlarge the gas–liquid interface and reduce the gas diffusion resistance in the liquid phase^8,10^.

Although gas separation efficiency is frequently emphasised, in the kinetic mechanism of hydrate growth, the diffusion and distribution of gas and water molecules does not receive equivalent focus, especially at the microscopic level. The micro-kinetic description of the preferential incorporation of CO_2_ in hydrate growth is typically obtained from macroscopic measurements and analysed by thermodynamic theory, but not fully verified at the microscopic level^3^. Because experiments on hydrate-based carbon capture are generally carried out in gas–liquid–hydrate systems, hydrate growth rates are estimated by calculating accumulated gas consumption without considering gas dissolution and the increasing hydrate nuclei, which may greatly affect hydrate formation^17,18^. In addition, molecular simulations and microscopic experiments have indicated that small gas molecules are active in hydrate formation^19,20^.

The major difficulties in measuring the micro-kinetics of hydrate growth are the growing hydrate surface and the determination of the gas molecules in the hydrate and liquid phases. Common methods allow hydrate particles to nucleate in the liquid phase, where the newly formed hydrate layer may quickly cover the original layer as the hydrate grows larger, inhibiting continuous tracking at a specific spot in the hydrate phase^21,22^. Meanwhile, high fluidity and adhesion may also change the growth pattern of hydrate particles. To overcome difficulties, ice has been chosen as a starting material because of its limited fluidity^23,24^. By observing the growth of hydrate on ice particles, Falenty et al.^25^ found that the newly formed hydrate layer grew outwards only by a few micrometres. These previous studies indicate that ice could be an ideal material for investigate the micro-kinetics of hydrate growth.

Therefore, in this study, hydrate formation from ice powder was measured in situ by Raman spectroscopy at 271.6 K. A simple method was proposed to characterise the growth of gas concentration in the hydrate phase. Synthesised flue gas, biogas, and pure CO_2_ were used to compare the gas adsorption rates of CH_4_, CO_2_, and N_2_ by hydrates. To verify the hydrate growth from the gas mixtures, macroscopic measurements were also performed under the same operating conditions as those in the Raman measurements (Table 1). The kinetics of hydrate formation were characterised according to gas enrichment in the hydrate phase. The preferential incorporation of gas molecules in different stages of hydrate formation was described quantitatively. The results provide a microscopic insight into the hydrate growth in the initial stage of hydrate growth, especially from gas mixtures.

**Table 1:** Initial conditions of in situ Raman measurements.

Starting material	Temperature (K)	Pressure (MPa)	Feed gas composition (mol%)		
			CO_2_	N_2_	CH_4_
Ih ice	271.6 ± 0.1	2.1 ± 0.15	99.9	–	–
Ih ice	271.6 ± 0.1	6.1 ± 0.15	20 ± 0.1	80 ± 0.1	–
Ih ice	271.6 ± 0.1	2.7 ± 0.15	40 ± 0.1	10 ± 0.1	50 ± 0.1

## Results

### Identification of characteristic spectra

**Figure 1:**
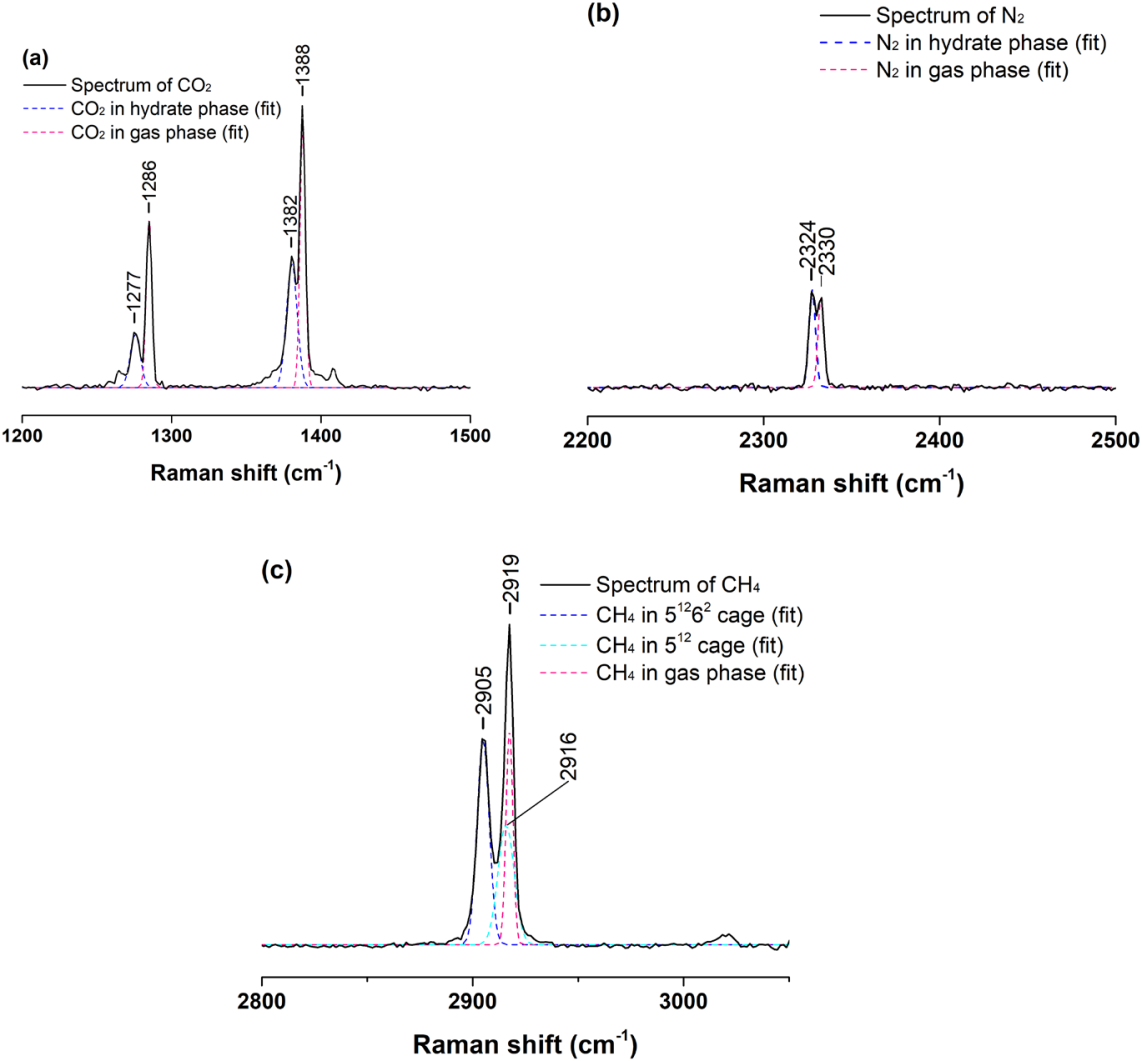
Typical spectra of CO_2_, N_2_, and CH_4_ observed from in situ Raman measurements at 271.6 K. The characteristic peaks of CO_2_, N_2_, and CH_4_ molecules in gas and hydrate phase are marked by dashed lines.

When the Raman laser focused on the hydrate surface, Raman peaks of gas molecules in both the gas and hydrate phases were observed, as shown in Fig. 1. The positions of the characteristic peaks of gases agreed well with the reference^26,27^. It should be noted that the Raman peak of gaseous CH_4_ at 2919 cm^−1^ overlapped with the peak of CH_4_ in the small cages of sI hydrates, which could not be clearly identified. Such a phenomenon could also be seen in the characteristic peaks of N_2_ molecules in the hydrate and gas phases. However, they could still be separated by the fitting curves, as shown in Figure S1. The peak of CH_4_ in the small cages of sI hydrates at 2916 cm^−1^ could be separated by fitting the peak at 2919 cm^−1^, while the peak of N_2_ in sI hydrates at 2324 cm^−1^ could be observed by fitting the peak at 2330 cm^−1^. In addition, no evident split was found to indicate the specific distributions of CO_2_ and N_2_ in the hydrate phase such that CO_2_ and N_2_ molecules in large and small cages could not be distinguished from the spectra^17^.

The Raman spectra of the O–H stretching modes of water molecules in Ih ice and sI hydrate are shown in Figure S1. Typically, the water band ranging from 2800 to 3800 cm^−1^ corresponds to symmetric and antisymmetric stretching vibrational modes of water molecules, accompanied by additional contributions from the Fermi resonance caused by the overlap of the O–H stretching mode frequency and the O–H bending overtone mode^28^. However, theoretical analysis revealed that the O–H stretching mode spectra of ice Ih were strongly affected by the vibrational modes coupled in a complex manner^29^. The spectral features in Ih ice were mainly determined by the large intermolecular couplings and diagonal disorder rather than intramolecular couplings^30–32^. By relating the frequency distributions to the spectra, four subpeaks could be observed, as shown in Figure S1. Interestingly, the shape of the water band did not noticeable change as Ih ice transformed into sI hydrate. In both Ih ice and sI hydrate, each water molecule was hydrogen-bonded to the four nearest neighbours in a tetrahedral arrangement, and the average H–O–H angle only departed a few degrees from the tetrahedral angle^33^. Therefore, the influence of the structural change from Ih ice to sI hydrate on the shape of the water bands was quite limited. Combining with the fact that the integrated peak intensities of gas and water molecules were proportional to their molar fractions in the hydrate phase^17,34^, quantitative descriptions of the gas concentrations in the hydrate phase were calculated by dividing the peak area of gases by the area of the water band.

### Hydrate formation from pure CO_2_

**Figure 2:**
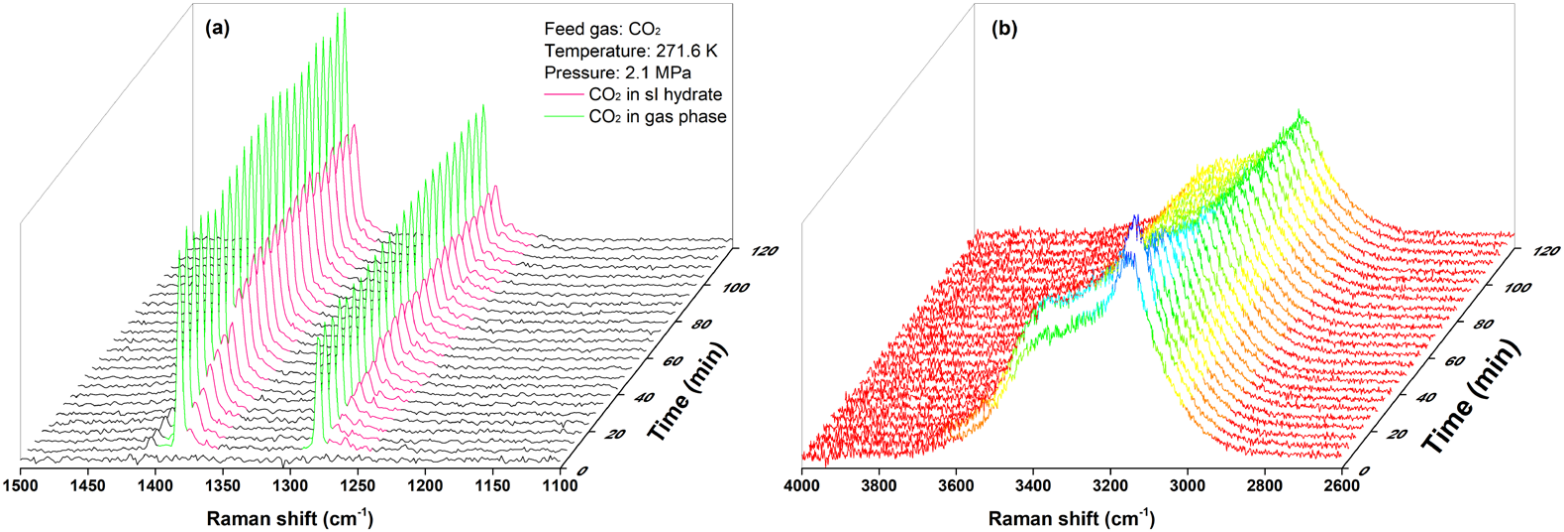
In situ Raman spectra of hydrate formation from pure CO_2_. (**a**) Peaks of CO_2_ molecules in hydrate and gas phase, (**b**) corresponding change of water band with colour mapping along intensity values.

Figure 2(a) shows the in situ Raman spectra of hydrate formation from pure CO_2_ at 271.6 K and 2.7 MPa. The peaks of gaseous CO_2_ remained almost constant through 120 min, while the peaks representing CO_2_ in the hydrate phase grew quickly in the initial 60 min and then gradually reached stability. This type of growth pattern was consistent with the previous observations and further indicated that the conversion from Ih ice to gas-saturated sI hydrate was a gradual process rather than an abrupt change^21,23,35^.

Another observed phenomenon was the gradual decrease in the intensities of the water bands, as seen in Fig. 2(b). In most of our experiments, the intensities of the water bands reduced by at least 20% of the initial values. The shift of the sample surface in the measurement could be the primary reason for this observation. Although the densities of Ih ice and sI hydrate did not deviate significantly, hydrate formation from Ih ice was still found to go through a volume increase associated with hydrate expansion^25^. Therefore, the laser spot originally focused on the sample surface was gradually embedded in the growing surface, which led to reductions in the peak intensities.

**Figure 3:**
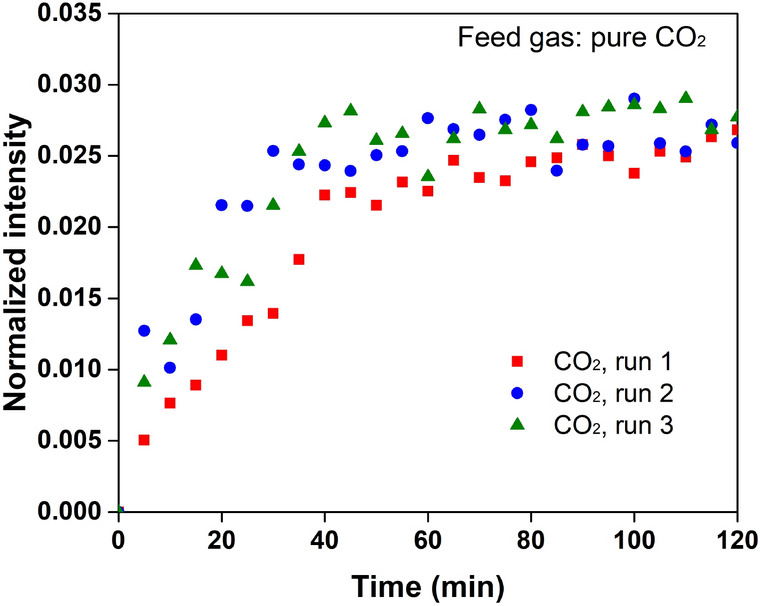
Growth of the normalized intensities of CO_2_ in the hydrate phase with time. Runs 1–3 are repeated tests performed at the same experimental conditions.

Profiles of the normalised intensity of CO_2_ in the hydrate phase are shown in Fig. 3. The profiles were found to follow a similar trend and gradually reached a stable value of approximately 0.029, indicating good repeatability of the experiment. The growth patterns of hydrate at each spot on the ice surface were generally the same. The normalised intensity of the encaged CO_2_ jumped to 0.009 at 5 min, which was 33% of the saturation state, and then increased gradually (Fig. 3). Structural conversion from Ih ice to sI hydrate was thought to take place once the gas was injected^25^. However, the newly formed hydrate nuclei seemed unsaturated with gas molecules. In other words, the water molecules formed the water lattice of sI hydrate first, and then the unsaturated hydrate structure was stabilised via continuous gas adsorption. Another possible explanation for this increase was that the Ih ice at the measuring spots was not fully converted into hydrate. However, the thickness of the formed hydrate film was at least 2 μm, which is larger than the size of the measuring spot, according to previous studies^23^. In this case, the newly formed hydrates were assumed to be in an unsaturated and unstable state. They continuously adsorbed gas molecules until a stable state was reached.

The gas saturation at hydrate nucleation has not been well understood. Newly formed hydrate nuclei are usually assumed to be in an equilibrium state in classical nucleation theory^9,11^. Simulation results have also indicated that the cage occupancies of gases in the newly formed hydrate layer have to reach 90% to guarantee the stability of the hydrate structure^8,36^. However, most of these tests allowed hydrates to grow in liquid water where water molecules were mobile and abundant. More importantly, the structural order of the water lattice can be easily changed in liquid water, especially with the help of gas molecules. In the conversion from ice to hydrate, most of the water molecules were hydrogen-bonded in a tetrahedral arrangement. As gas molecules penetrated into the Ih ice phase, the structural type was not easily changed to accommodate gas molecules while keeping the water molecules tetrahedrally hydrogen-bonded. The slow gas diffusion was also limited by the gas concentration at the recrystallization front of water molecules. The unsaturated hydrate phase, which was stabilised by only a few gas molecules, was therefore formed at the beginning of hydrate growth^37,38^. In this case, hydrate formation from Ih ice could be viewed as a formation of hydrate nuclei followed by continuous gas adsorption.

### Hydrate formation from synthesized flue gas

In the hydrate formation from the synthesised flue gas, the growth pattern of the CO_2_ peaks representing the encaged CO_2_ was generally the same as that in CO_2_ hydrate growth, as seen in Figure S2. Characteristic peaks of CO_2_ in the sI hydrate phase were found at 5 min, verifying the importance of CO_2_ molecules in the structural determination and stabilisation of the formed hydrates. The characteristic peaks of the encaged N_2_ could not be directly observed from the in situ spectra due to the high overlap of the Raman peaks of gaseous and encaged N_2_ molecules. To quantitatively analyse the N_2_ concentration in the hydrate phase, the peaks of N_2_ in sI hydrates at 2324 cm^−1^ were separated by the fitting curves, as previously mentioned.

The growth patterns of the normalised intensities of CO_2_ and N_2_ molecules in the hydrate cell were different. In the formation of hydrate nuclei, the normalised intensity of CO_2_ in the hydrate phase jumped to 0.003 at 5 min, which was about 30% of the saturation state, and then increased gradually. The normalised intensity of N_2_ also jumped to approximately 0.003 at the start of hydrate growth but remained stable thereafter, as seen in Figure S3. In the synthesised flue gas where N_2_ was abundant, a considerable amount of gas molecules was assumed to be required after the formation of the unsaturated hydrate structure. However, the N_2_ concentration in the hydrate phase reached saturation once the hydrate crystallised, suggesting that N_2_ molecules were more active than CO_2_ in the formation of hydrate nuclei. This may be closely related to the small size of N_2_ molecules, which are weak in cage stabilisation but strong in cage penetration^19,36^. As for CO_2_, the presence of CO_2_ prevented N_2_ to form sII hydrate and allowed the hydrate growth to take place at lower pressure so that the enrichment of CO_2_ in the hydrate phase followed the subsequent gas adsorption process^17,39^.

**Figure 4:**
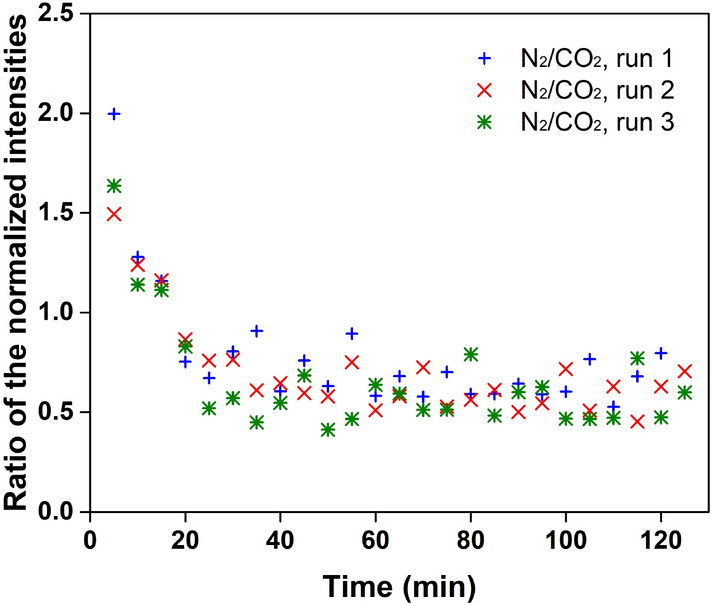
The ratio of the normalized intensities of N_2_ and CO_2_ molecules in hydrate formation from synthesized flue gas.

Figure 4 shows the preferential incorporation of N_2_ molecules in the initial stage. The ratio of the normalized intensities of N_2_ and CO_2_ declined continuously from about 1.7–0.6 in the first 30 min. It should be noted that the ratio of the normalized intensities of N_2_ and CO_2_ did not reflect the absolute concentration ratio of N_2_ and CO_2_ in the hydrate phase, but it indicated the concentration growth of CO_2_ in the hydrate phase was more evident than that of N_2_ during hydrate crystallization. Since the N_2_ concentration in the hydrate phase had already reached saturation in the formation of hydrate nuclei, the decrease of the ratio of the normalized intensities of N_2_ to CO_2_ does not mean that the increase of N_2_ concentration in the hydrate phase was slower than that of CO_2_. On the contrary, the slow adsorption of CO_2_ is suggested to be a rate-limiting step^19^. From another perspective, the abundance of N_2_ molecules in newly formed hydrate nuclei increased the ratio and resulted in the evident decrease in the ratio during gas adsorption. In this case, N_2_ molecules were essential to the formation of hydrate nuclei.

### Hydrate formation from synthesized biogas

In the hydrate formation from synthesised biogas, the kinetic properties of CO_2_ during cage filling were also found to be less efficient than those of CH_4_ molecules. The N_2_ peaks were greatly reduced because of the low N_2_ composition in the feed gas (Figure S4), and the characteristic peaks of the encaged N_2_ molecules were difficult to separate by the fitting curves so that the amount of N_2_ molecules in the hydrate phase was negligible. The growth patterns of the Raman peaks of CO_2_ and CH_4_ in the hydrate phase were generally the same. The competitive incorporation of gas molecules for hydrate growth was therefore thought to occur between CH_4_ and CO_2_.

In contrast to the characteristic peak of N_2_ in the hydrate phase, the peaks of CH_4_ in the large (5^12^6^2^) and small (5^12^) cages could be distinguished in the spectra. A more specific distribution of CH_4_ in the hydrate phase was thus obtained, as shown in Figure S5. At 5 min, the normalised intensities of CH_4_ in the large and small cages jumped to 0.003 and 0.006, which were approximately 29% and 65% of the saturation state, respectively. The CH_4_ concentrations in the small cages were two times higher than those in the large cages at the start of hydrate growth. CH_4_ molecules were preferably adsorbed in the small cages because of their suitable molecular size^6,8^, but were replaced by CO_2_ molecules in the large cages.

**Figure 5:**
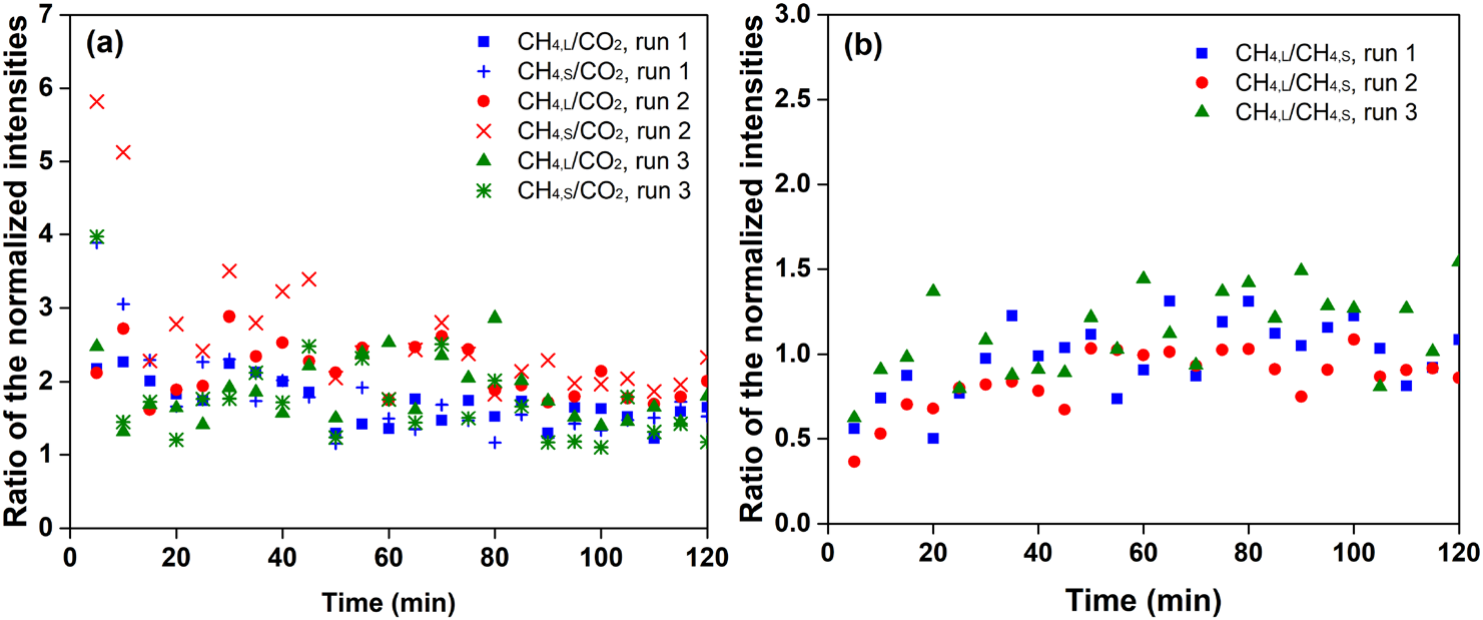
The ratio of the normalized intensities of CH_4_ and CO_2_ molecules and the CH_4_ in the large and small cages in hydrate formation from synthesized biogas. The normalized intensities of CH_4_ in large and small cages are labelled as CH_4,L_ and CH_4,S_, respectively.

The competitive occupation between CH_4_ and CO_2_ was found to behave differently during hydrate formation. When hydrate nuclei were newly formed on the ice surface, the normalised intensity of CO_2_ in the hydrate phase was 0.001 at 5 min, which was approximately 23% of the saturation state, as shown in Figure S5. Compared with hydrate formation from pure CO_2_ and synthesised flue gas, the initial normalised intensities of CO_2_ in the hydrate phase were found to decrease from 33 to 23% of their values at the saturation state. In addition, the ratio of the normalised intensities of CH_4_ and CO_2_ was decreasing as seen in Fig. 5(a). N_2_ and CH_4_ molecules were thought to exhibit growing importance in stabilising the unsaturated hydrate structure at the beginning of hydrate formation, and CH_4_ molecules performed better than N_2_ molecules.

During hydrate growth, the normalised intensities of CH_4_ in the large and small cages continued to grow, and the ratio of these intensities was found to increase from approximately 0.5–1.2, as seen in Fig. 5(b). Since two thirds of the CH_4_ molecules were incorporated in the small cages at the beginning of hydrate formation, CH_4_ molecules appear to be important in stabilising the small cages of the hydrate nuclei. However, in the subsequent gas adsorption period, the growth rates of CH_4_ in the large cages were higher than those in the small cages, indicating that the difficulty of CH_4_ entering the small cages was higher than that in the large cages.

In the subsequent gas adsorption period, the concentrations of both CH_4_ and CO_2_ continued to grow, which is known as a competitive cage occupation process^40^. The ratio of CH_4_ and CO_2_ in large cages decreased slowly from approximately 2.3–1.8, while the ratio of CH_4_ and CO_2_ in the small cages dropped from approximately 4.6–2.5 within 30 min, as shown in Figure S4. The CO_2_ concentration in the hydrate phase were posited to be grow faster than the CH_4_ concentration in both large and small cages, which was in accordance with the previous conclusion that CO_2_ molecules were preferentially incorporated in the formation of mixed CH_4_–CO_2_ hydrates^40^.

Based on the above analysis, two kinetic stages could be distinguished in the hydrate formation from Ih ice. The first stage was the formation of unsaturated hydrate nuclei, which were completed within the initial 5 min of in situ Raman measurements. The second stage was the continuous adsorption of gas molecules, which was evident from the growth of the normalised intensities of gas molecules in the hydrate phase. However, the enrichment of gas molecules in the first stage was found to be different in this study. CO_2_ molecules did not occupy most of the cages in the hydrate nuclei. Instead, small molecules such as N_2_ and CH_4_ behaved more actively than CO_2_ during the formation of hydrate nuclei. While the mechanism of this phenomenon remains unclear, the results revealed the importance of small gas molecules in hydrate formation from gas mixtures.

### Macroscopic measurements

**Figure 6:**
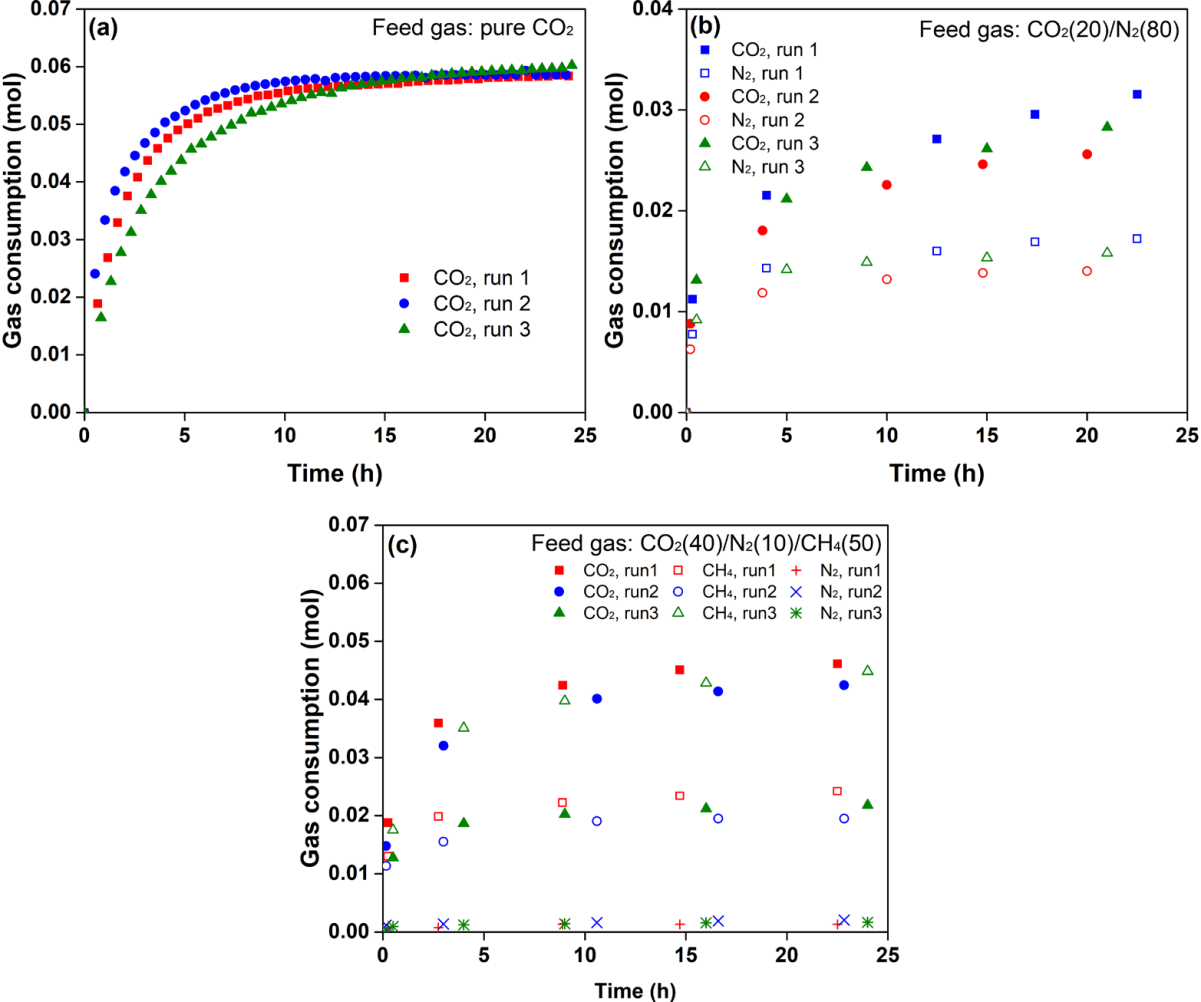
Profiles of the accumulated gas consumption in hydrate formation from (**a**) pure CO_2_, (**b**) flue gas, and (**c**) biogas.

Figure 6 shows the gas consumption during 25 h of hydrate formation. The repeated tests indicated that the results from each experiment had good consistency. In the hydrate formation from pure CO_2_, the CO_2_ consumption profiles were generally the same as the growth pattern of the normalised integrated intensities of CO_2_ in Raman measurements. In the initial 12 h, the accumulated CO_2_ consumption grew quickly, and the gas consumption rate was assumed to be primarily limited by the hydrate growth on the surface of the ice powder^3^. After the initial 12 h, CO_2_ consumption slowed over time, although the accumulated CO_2_ consumption was approximately 0.059 mol, which was far less than the amount of CO_2_ required for the complete conversion of ice into hydrate (0.179 mol). Therefore, the gas consumption after 12 h was largely limited by the gas diffusion in the hydrate phase. Similar to the sharp increase in the normalised intensity of the encaged CO_2_ at 5 min, the CO_2_ consumption also jumped to approximately 0.02 mol at 30 min. The formation of hydrate nuclei was assumed to occur once the pressure surpassed the equilibrium pressure of CO_2_ hydrate.

**Figure 7:**
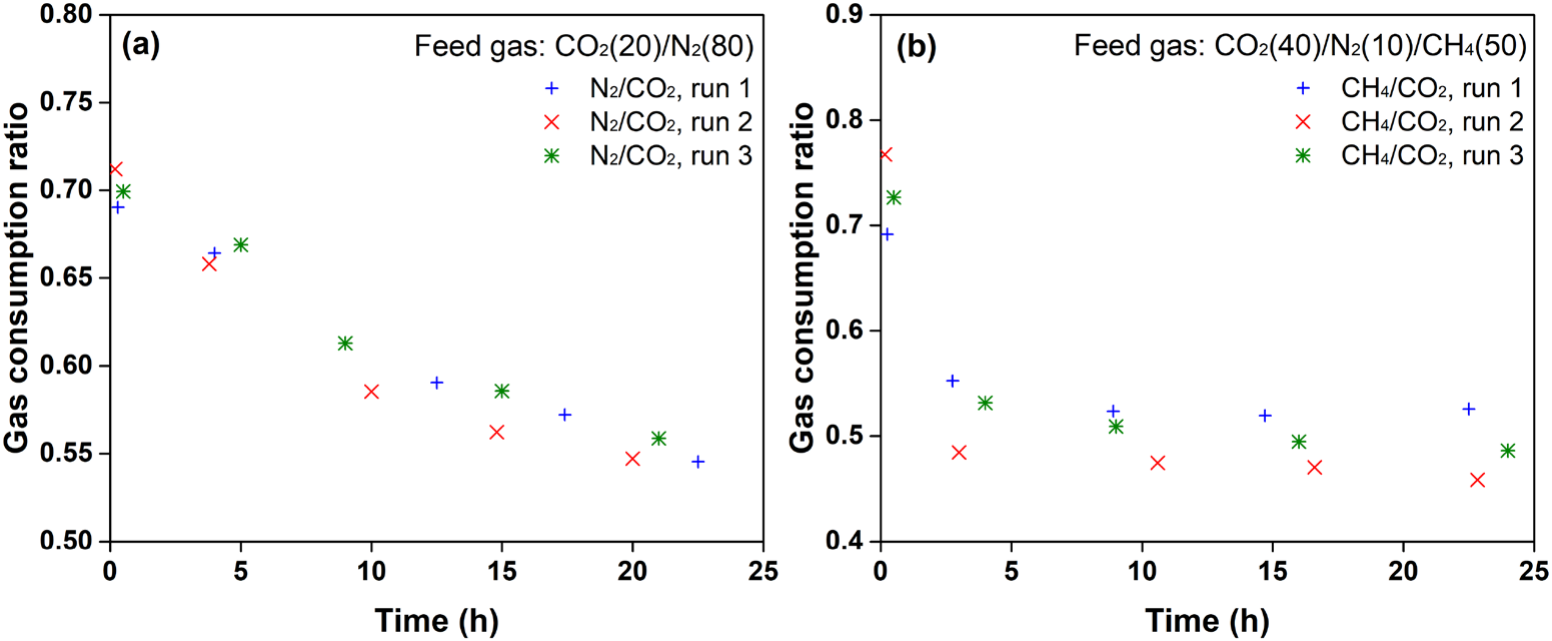
The gas consumption ratio of N_2_ to CO_2_ and CH_4_ to CO_2_ in the hydrate formation from flue gas and biogas, respectively.

In the hydrate formation from flue gas, a sharp increase in CO_2_ and N_2_ consumption at 30 min could also be observed. Then, CO_2_ was consumed almost linearly with time while N_2_ consumption grew much slower. The ratio of N_2_ to CO_2_ consumption decreased from 0.70 to 0.55 (Fig. 7), suggesting a strong demand for CO_2_ in hydrate growth. It also indicated that N_2_ molecules were more active than CO_2_ molecules in the formation of hydrate nuclei, which is consistent with the conclusion obtained from in situ Raman measurements.

In the hydrate formation from biogas, N_2_ molecules were shown to be less involved in hydrate growth, as seen in Fig. 6(c). Competitive adsorption of CH_4_ and CO_2_ dominated hydrate growth. The amount of CO_2_ adsorbed was twice that of CH_4_, although the mole percent of CH_4_ was relatively higher than that of CO_2_ in the feed gas. CO_2_ molecules were more preferentially adsorbed by the hydrates. However, CH_4_ molecules behaved more actively than CO_2_ molecules at the initial stage. The ratio of CH_4_ to CO_2_ was found to be around 0.73, as seen in Fig. 7(b), suggesting that CH_4_ molecules also played a key role in promoting the nucleation of hydrates.

## Discussion

To reveal the kinetic performance of gas molecules in hydrate growth, hydrate formation from pure CO_2_, flue gas, and biogas was measured by in situ Raman spectroscopy and a macroscopic method at 271.6 K. Hydrate formation from Ih ice was found to initiate with hydrate nucleation followed by continuous gas adsorption. Gas molecules were found to have the same kinetic behaviour in both in situ Raman and macroscopic measurements. CO_2_ molecules were preferentially adsorbed in the hydrate phase. The gas concentrations in the hydrate phase were observed to have a sharp rise at 5 min, and the initial normalised intensities of CO_2_ in the hydrate phase were 23–33% of their values at saturation states. In the subsequent process, CO_2_ adsorption was also more evident than that of CH_4_ and N_2_ molecules. However, CH_4_ and N_2_ molecules were more active in the initial stage of hydrate formation. The ratios of N_2_ to CO_2_ and CH_4_ to CO_2_ were found to decrease during hydrate growth in both in situ Raman and macroscopic measurements. In this case, small molecules such as N_2_ and CO_2_ were suggested to be more active in forming unsaturated hydrate nuclei, while the preferential incorporation of CO_2_ molecules took place in the subsequent gas adsorption process. In the following studies, we will focus on the effect of temperature and pressure on the selective absorption of hydrate at the initial stage of hydrate growth.

## Methods

The hydrate formation from Ih ice were measured at 271.6 K using in situ Raman and macroscopic methods. In macroscopic measurements, hydrates were formed from ice powder which was densely packed in a cylinder shape. Gas samples were taken at regular time intervals as seen in Fig. 6 and analysed by a gas chromatography. In situ Raman measurements were performed in a high pressure optical reactor. The laser spot was fixed at the ice surface to obtain the spectral change from ice to hydrate. Information about the devices and procedures of was detailed in supporting information.

## Supplementary Information

Supplementary Information 1.
