# Supplementary material for: Multiscale analysis of the hydrate based carbon capture from gas mixtures containing carbon dioxide
Source: Sci Rep. 2021 Apr 28;11:9197. doi: 10.1038/s41598-021-88531-x (PMC8080785; doi:10.1038/s41598-021-88531-x)

**Electronic Supporting Information**

**“Multiscale analysis of the hydrate based carbon capture from gas mixtures containing carbon dioxide”**

by Xuebing Zhou, Xiaoya Zang, Zhen Long, and Deqing Liang

**Items:**

- Supporting Methods and Materials
- Supporting Figure S1-S5

Supporting methods and materials

In microscopic measurements, an in situ Raman device was used which equipped with a custom designed high-pressure optical reactor made of stainless steel. The inner chamber was sealed with a 6 mm thick sapphire glass from the top and could withstand 12 MPa. A brass stage inside the chamber was used to hold the sample, and the chamber had a thermal resistance with an accuracy of ± 0.01 K, which was used to measure the sample temperature. The sample temperature was controlled by a thermostatic bath with a precision of ± 0.1 K. Two high-pressure tanks were connected to the inlet and outlet of the chamber, which were used to stabilise the pressure with a precision of ± 0.01 MPa. More information about the equipment can be found in our previous work^1^.

The confocal Raman spectrometer was equipped with a 2400 grooves/mm grating and multichannel air-cooled charge-couple device detector. An Ar laser beam (wavelength, 532 nm; power, 50 mW) was irradiated from the object lens (Olympus 50×) and condensed to a 1 μm circular spot on the hydrate surface. Standard silicon crystals were used for calibration. The spectrum scanning range was from 100 to 4000 cm^-1^ with a spectral resolution of approximately 1.4 cm^-1^ and the acquisition time was 135 s.

The powder of Ih ice was chosen as the starting material for hydrate formation. Double-distilled water (resistivity, 18 MΩ/cm^-1^) was first frozen in a refrigerator. Then, the ice blocks were finely ground in liquid nitrogen. As the optical reactor was cooled down to 271.6 K, the brass stage was removed from the reactor to load the ice powder. After the temperature in the chamber remained stable, the optical reactor was sealed and fixed on the X-Y stage of the Raman spectrometer. The objective lens was then adjusted to a suitable spot on the ice surface where the broad band of water molecules ranging from 2800 to 3800 cm^-1^ could be clearly observed. Then, the precooled feed gas in the high-pressure tank was slowly loaded into the optical reactor to sweep the air in the chamber and increase the pressure. All the in situ Raman tests were carried out at 271.6 K with constant pressure, which was approximately 1 MPa above the equilibrium pressure of the formed hydrates (Table 1). In each test, the measuring spot was fixed, and the Raman spectra were recorded every 5 min.

As the water bands of Ih ice and sI hydrate did not change evidently, a quantitative method to measure hydrate growth was used, where water bands were treated as reference peaks. Specifically, the intensities of the characteristic peaks of CO_2_ at 1382 cm^-1^, N_2_ at 2324 cm^-1^, and CH_4_ at 2905 and 2916 cm^-1^, and a water band ranging from 2850 cm^-1^ to 3800 cm^-1^ were integrated. This allowed the calculation of the normalised integrated intensities of gases by dividing the peak area of gases by the area of the water band, which gave quantitative descriptions of the gas concentrations in the hydrate phase^2,3^. This data processing method is based on the fact that the integrated peak intensities of gas and water molecules were proportional to their molar fractions in the hydrate phase, and that the shape of the water bands did not change noticeably during the conversion from Ih ice to sI hydrate^1,4^. Therefore, choosing water bands as reference peaks provides a path to compare the peak increase of gas molecules in different experiments.

Macroscopic measurements were taken in a 158 mL high-pressure reactor equipped with a 1.5 L gas reservoir. The thermostatic bath was used to control the temperature of the reactor and gas reservoir in the range of 243.15 to 323.15 K. The thermodynamic conditions of the reactor and gas reservoir were monitored by a thermal resistance and a pressure transducer with an accuracy of ± 0.1 K and 0.01 MPa, respectively. The gas sample was determined using a gas chromatograph connected to the reactor. A detailed description of the experimental device can be found in our previous work^5^.

Before hydrate formation, Ih ice was finely ground in liquid nitrogen and densely packed in a cylinder shape, which was 40 mm in length, 27 mm in diameter, and 18.5 ±0.2 g in mass. The ice powder was densely packed to increase the specific surface of the Ih ice and avoid the melting of the ice powder during gas injection. After the reactor was chilled to approximately 233 K, the ice powder was placed in the reactor. The reactor was then connected to the gas reservoir and evacuated for 15 min. Finally, the reactor was quickly loaded with hydrate-forming gas and then immersed in a thermostatic bath. The running conditions of each experiment were the same as those in the in situ Raman measurements, as shown in Table 1. To enhance the selective absorption of different gases in hydrate growth, we chose to measure hydrate formation in isochoric system without changing the experimental conditions significantly. In isochoric system, no additional feed gas were added so that the change of gas composition in gas phase was more evident than isobaric system and the gas consumed for hydrate formation could be calculated accurately. To control the loss of driving force in hydrate formation. The pressure drop during hydrate formation were controlled within 0.3 MPa including the pressure drop caused by gas sampling so that the loss of driving force was less than 30%. Gas samples were taken at regular time intervals. The gas consumption of hydrate formation was calculated using PR Eos^6,7^. To guarantee repeatability, each experiment was repeated three times.

*References*

1. Zhou, X. B., Lin, F. H. & Liang, D. Q. Multiscale analysis on CH_4_-CO_2_ swapping phenomenon occurred in hydrates. *J. Phys. Chem. C* **120,** 25668-25677 (2016).

2. Chazallon, B. & Pirim, C. Selectivity and CO_2_ capture efficiency in CO_2_-N_2_ clathrate hydrates investigated by in-situ Raman spectroscopy. *Chem. Eng. J.* **342,** 171-183 (2018).

3. Qin, J. F. & Kuhs, W. F. Quantitative analysis of gas hydrates using Raman spectroscopy. *AIChE J.* **59,** 2155-2167 (2013).

4. Xu, C. G. et al. Insight into micro-mechanism of hydrate-based methane recovery and carbon dioxide capture from methane-carbon dioxide gas mixtures with thermal characterization. *Appl. Energ.* **239,** 57-69 (2019).

5. Zhou, X. B. et al. In situ Raman analysis on the dissociation behavior of mixed CH_4_-CO_2_ hydrates. *Energ. Fuel* **30,** 1279-1286 (2016).

6. Fateen, S. E., Khalil, M. M. & Elnabawy, A. O. Semi-empirical correlation for binary interaction parameters of the Peng-Robinson equation of state with the van der Waals mixing rules for the prediction of high-pressure vapor-liquid equilibrium. *J. Adv. Res.* **4,** 137-45 (2013).

7. Hu, P., Chen, L. X. & Chen, Z. S. A modified differential-model for interaction parameters in PR EoS with vdW mixing rules for mixtures containing HFCs and HCs. *Fluid Phase Equilibr.* **324,** 64-69 (2012).

- Figure S1. Raman spectra of water bands obtained before and after hydrate growth. The dashed lines are four subpeaks which indicate contributions from constructive interference of many in-phase transitions, strong intermolecular couplings at low frequency, weak intermolecular couplings, and strong intermolecular couplings at high frequency, and are labelled as C, SL, W, and SH, respectively.


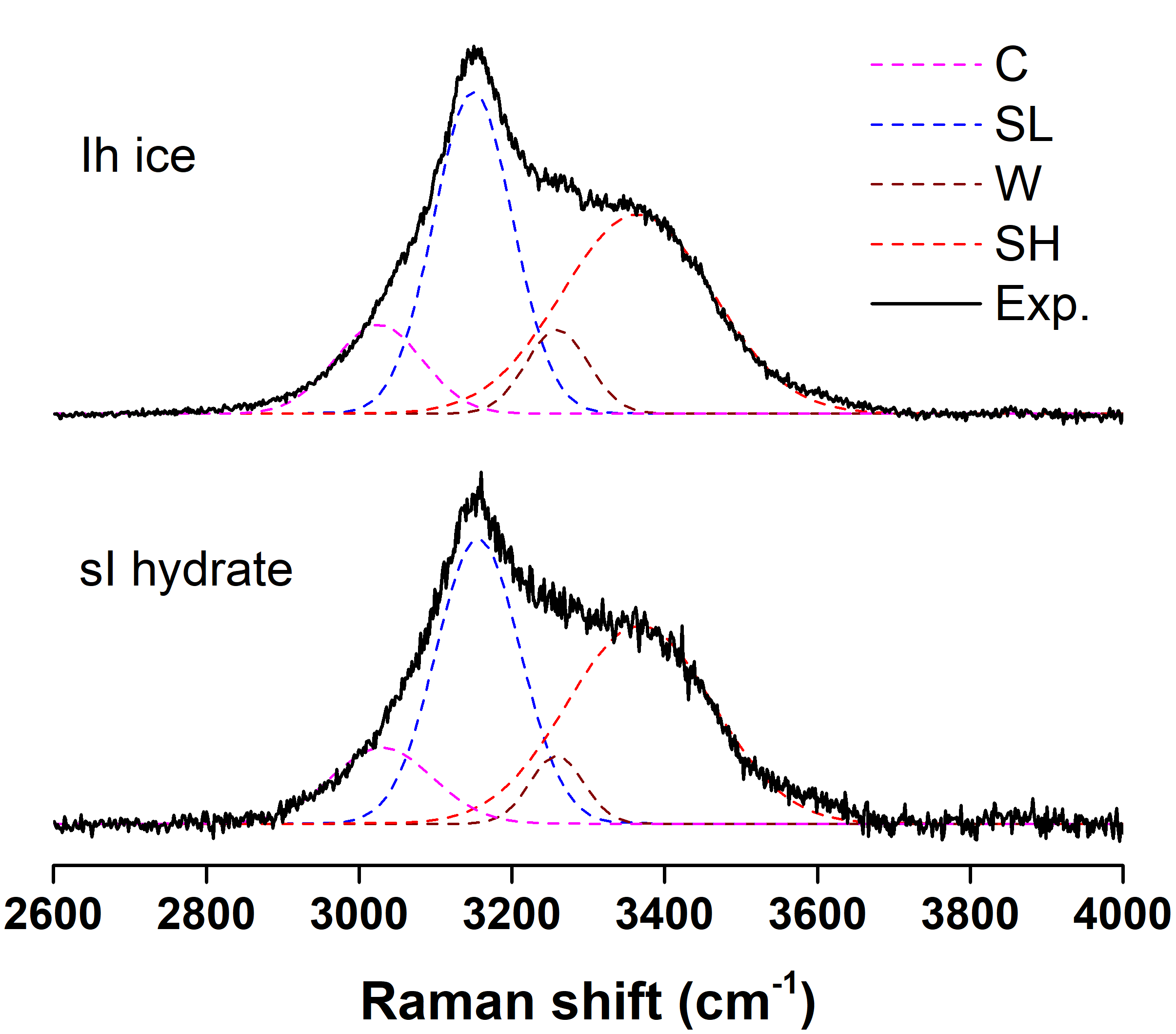


- Figure S2. In situ Raman spectra of hydrate formation from synthesized flue gas. (a) Peaks of CO_2_ molecules in hydrate and gas phase, (b) peaks of N_2_ molecules in hydrate and gas phase.


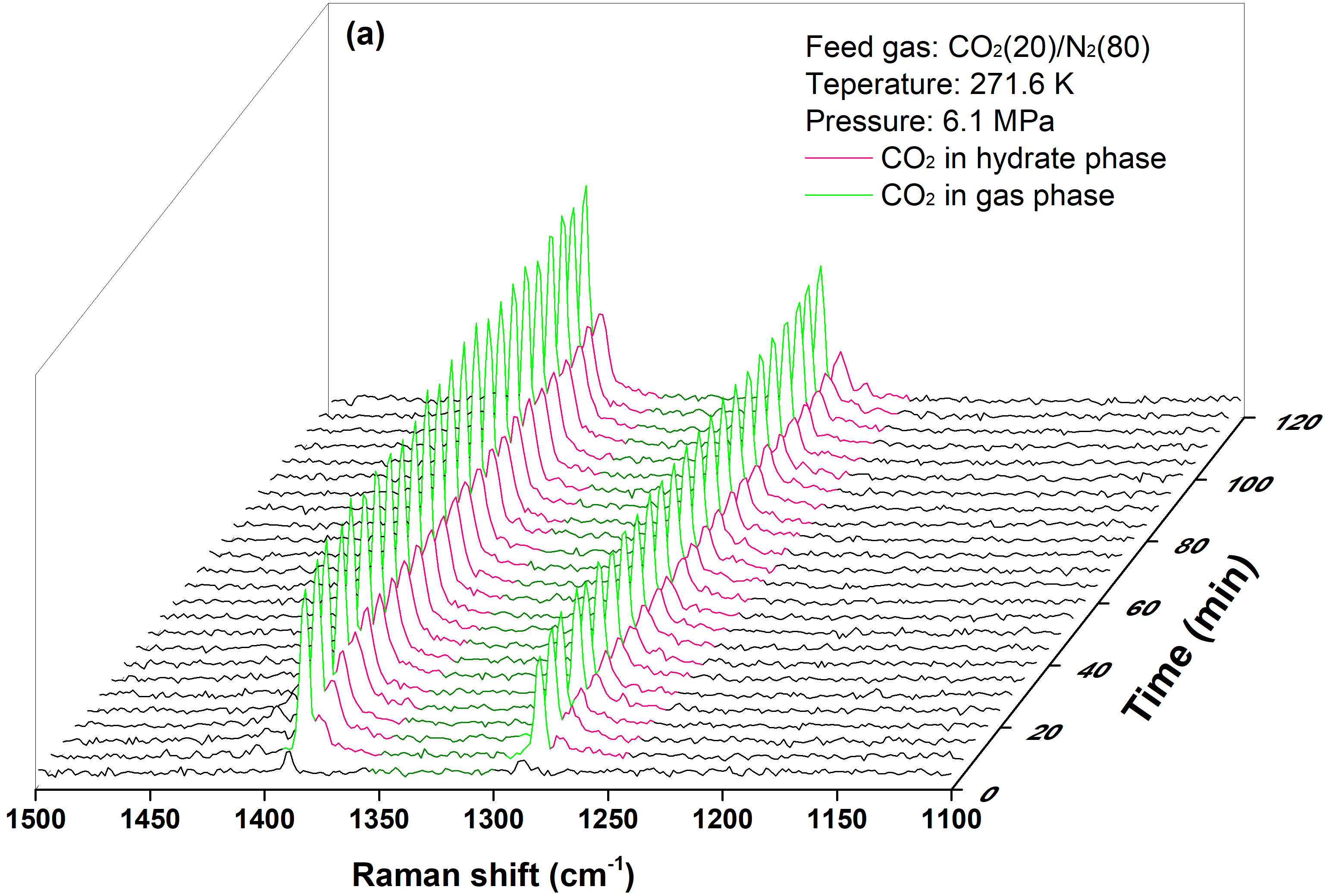

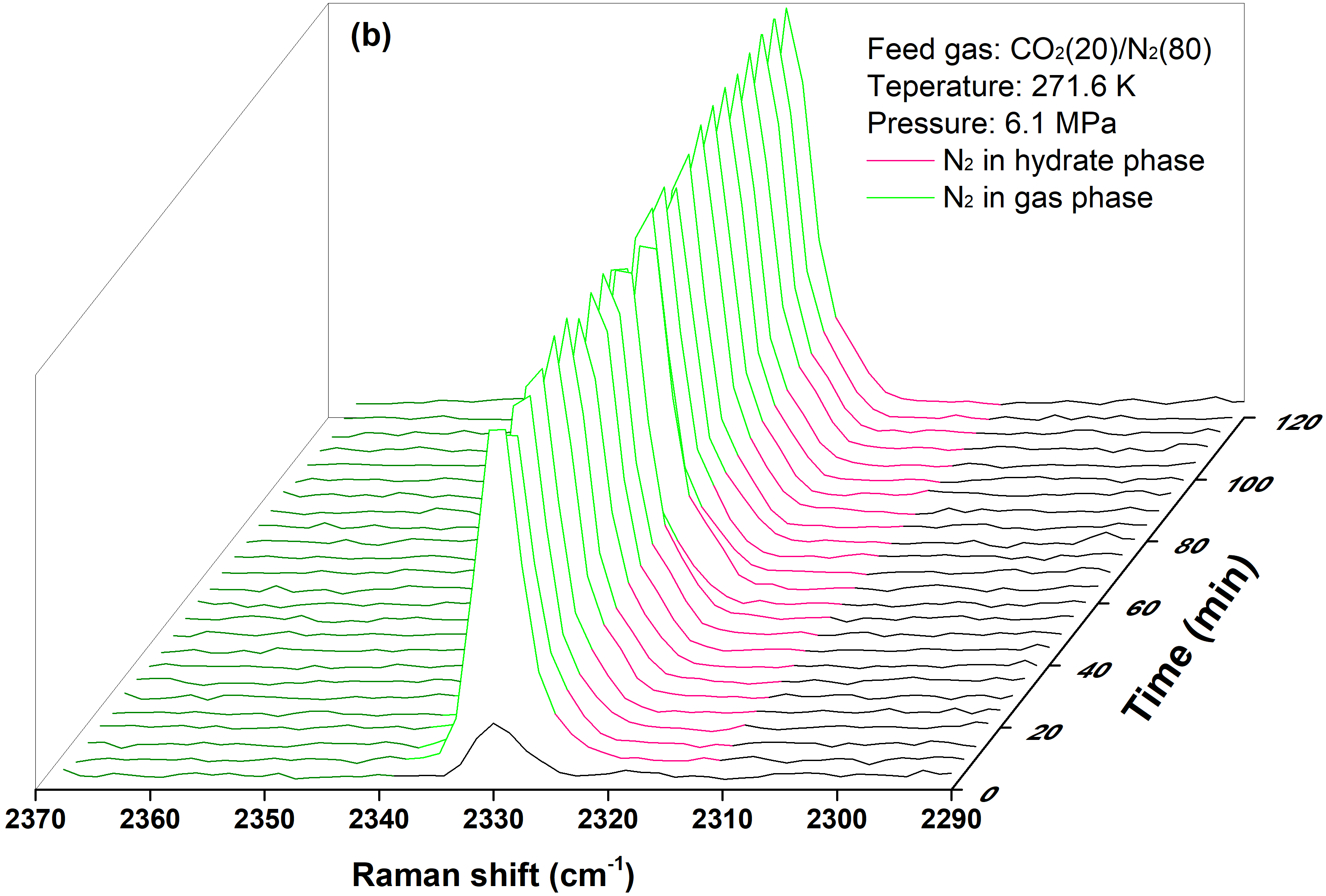


- Figure S3. Growth of the normalized intensities of CO_2_ and N_2_ in the hydrate phase with time. Runs 1–3 are repeated tests performed at the same experimental conditions.


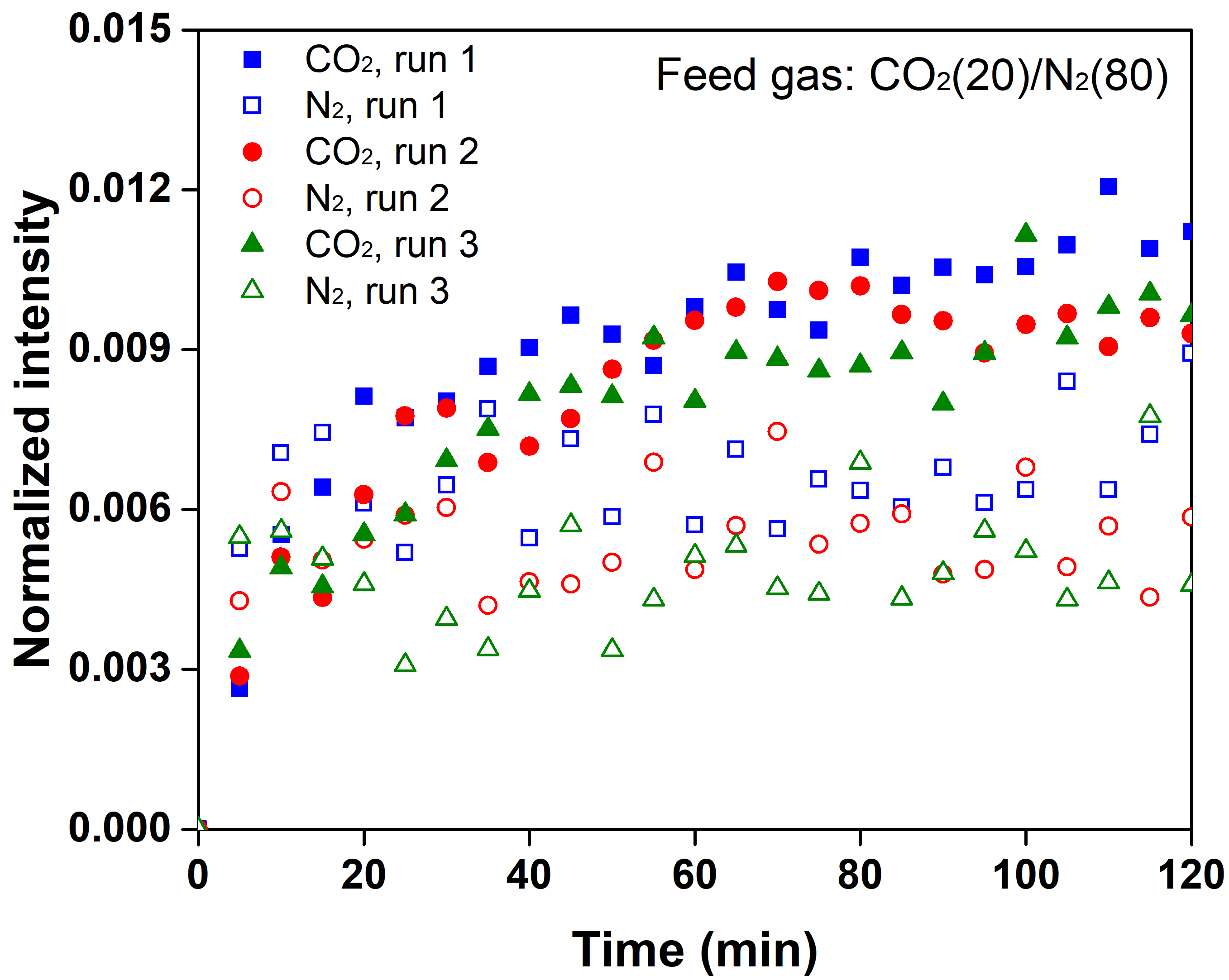


- Figure S4. In situ Raman spectra of hydrate formation from synthesized biogas.


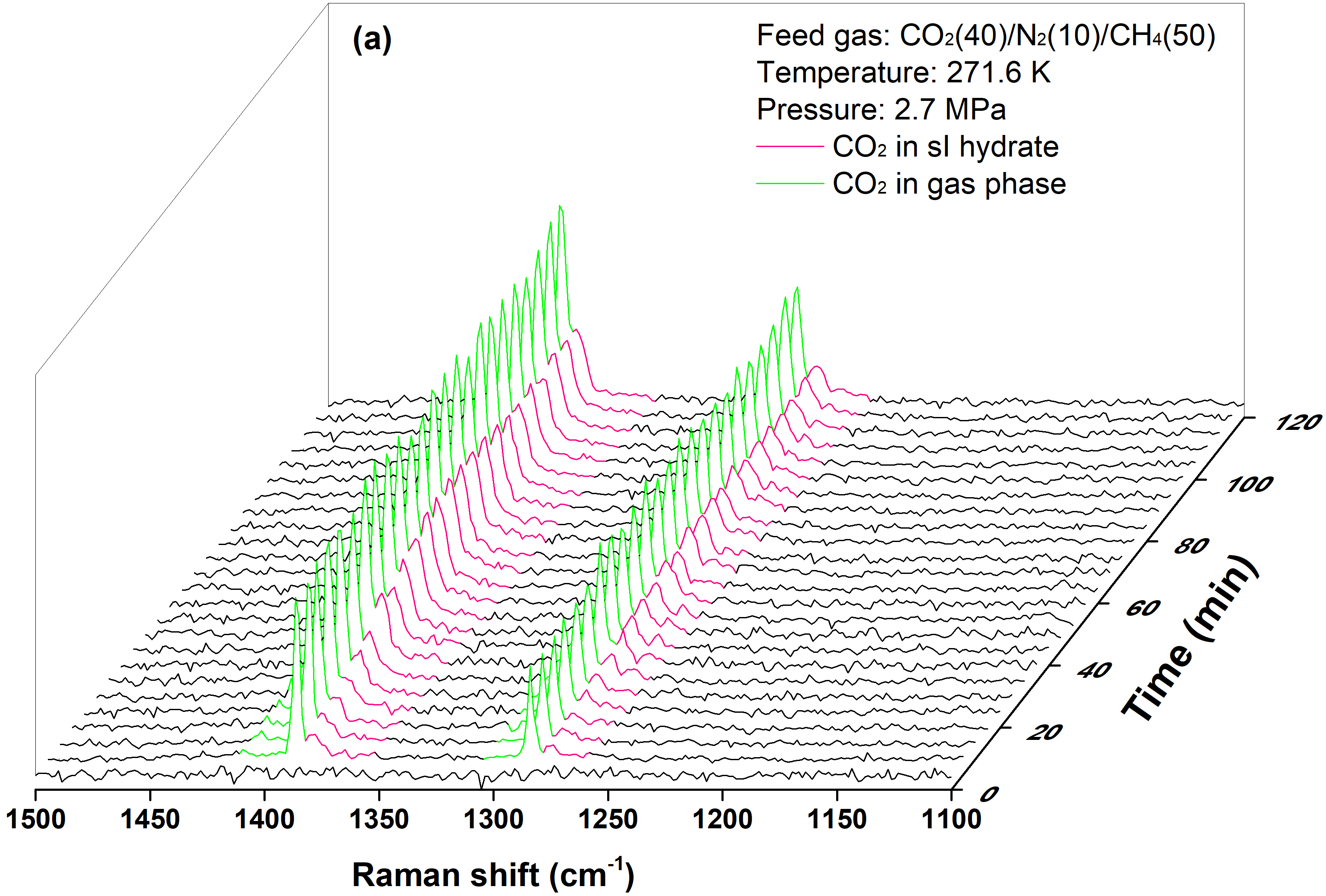

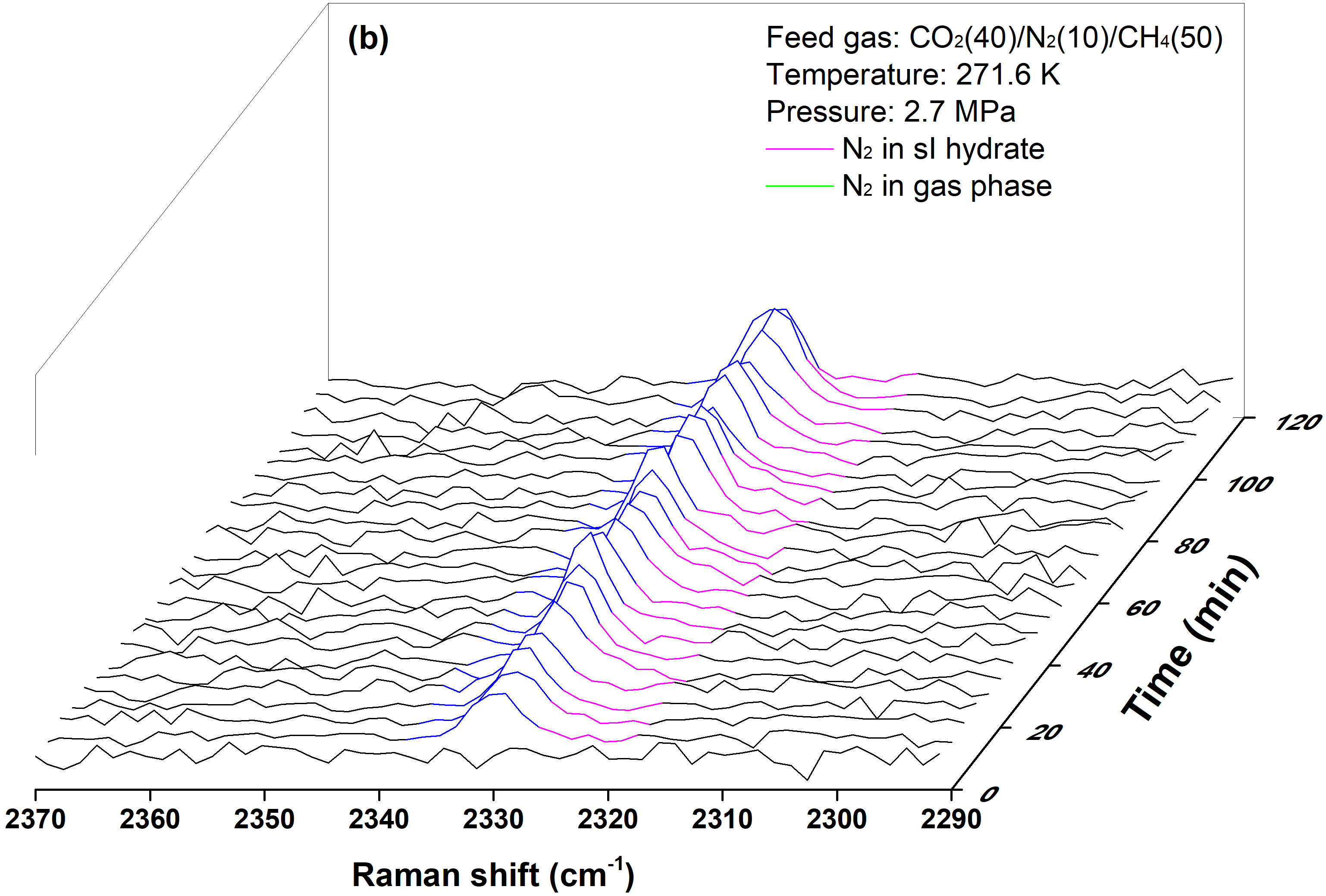


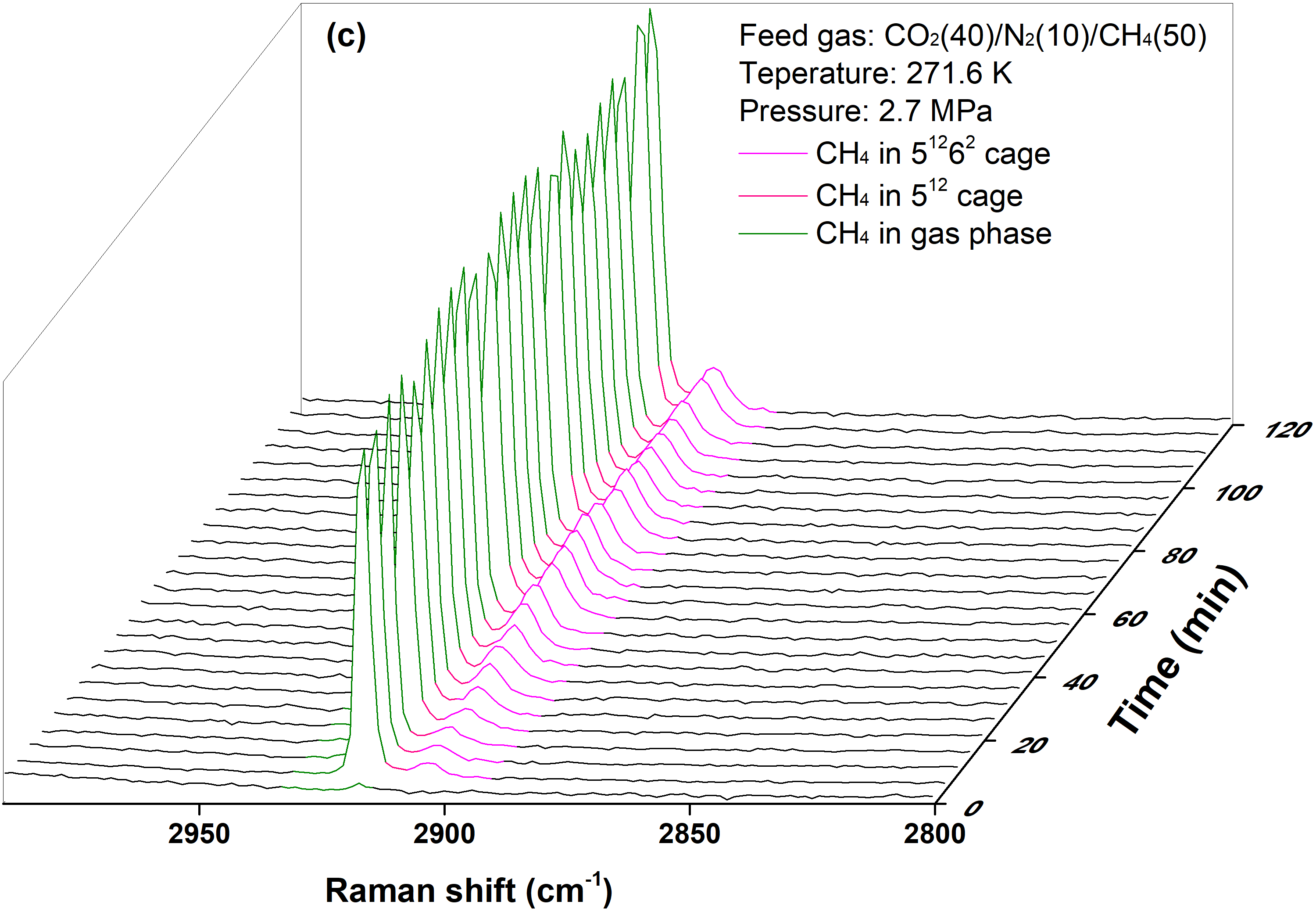


- Figure S5. Growth of the normalized intensities of CO_2_, N_2_ and CH_4_ in the hydrate phase with time. Runs 1–3 are repeated tests performed at the same experimental conditions.


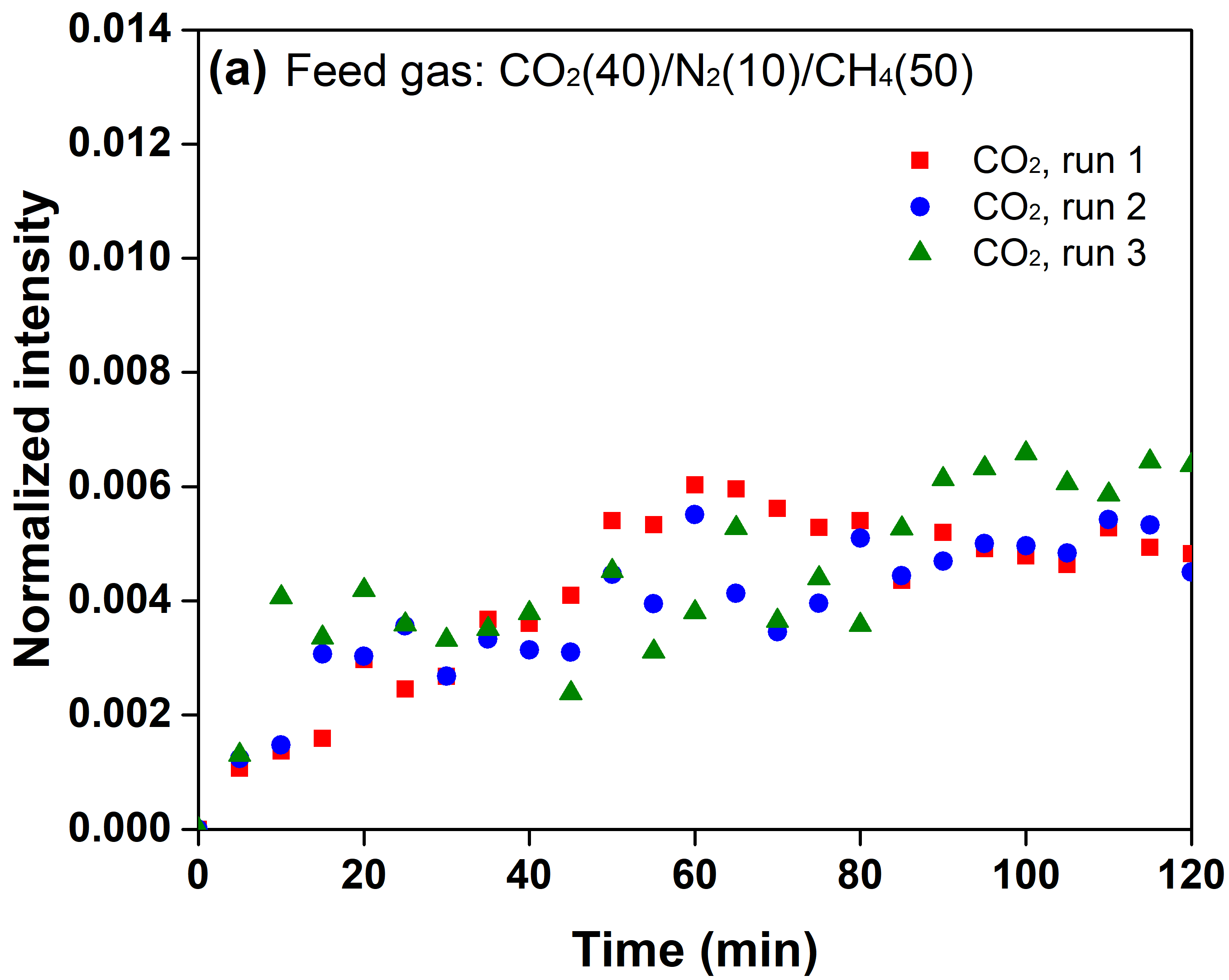

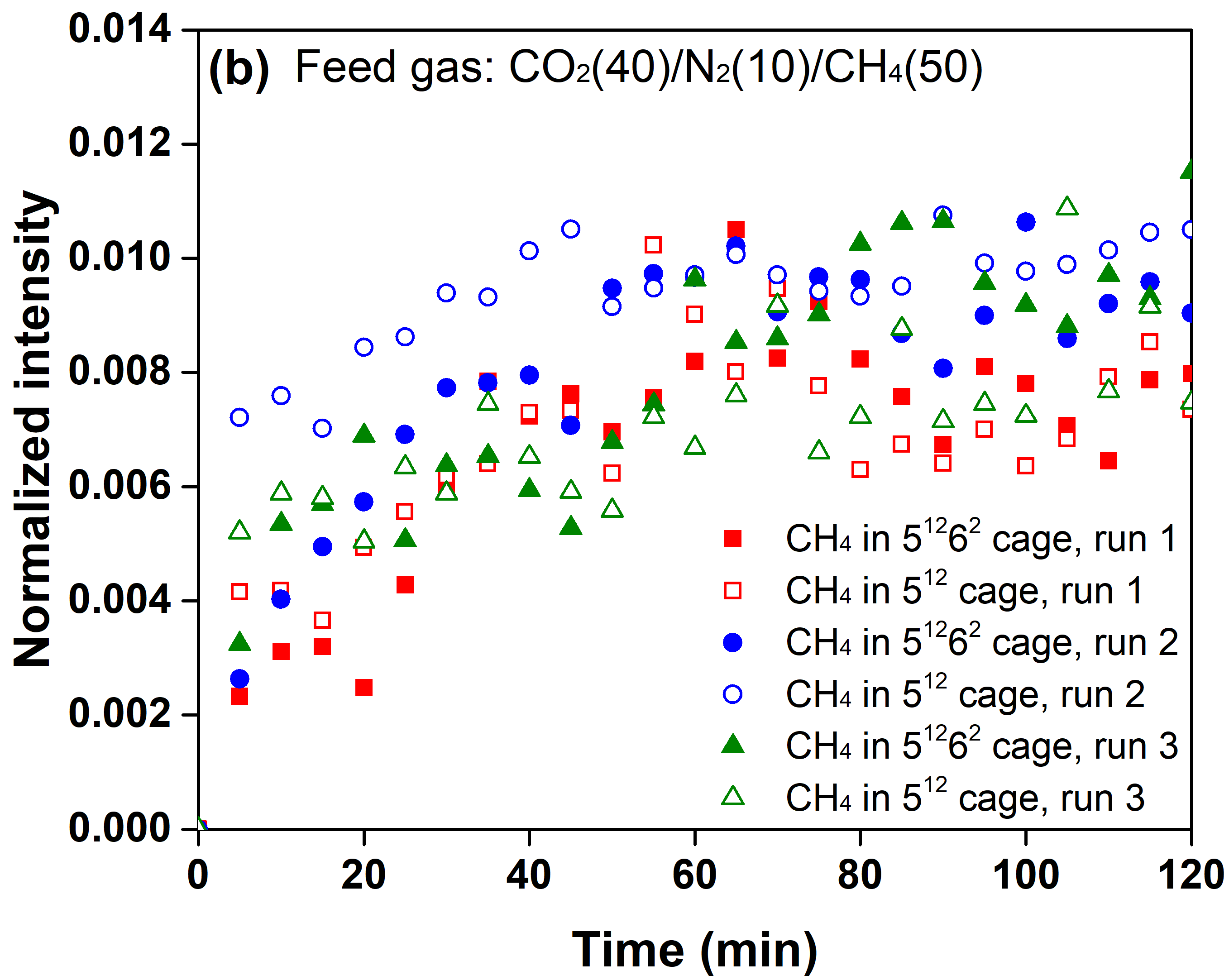

Supplement: Supplementary file 1 — Supplementary Information 1. [file 41598_2021_88531_MOESM1_ESM.docx]
